# Time course of changes in motor-cognitive exergame performances during task-specific training in patients with dementia: identification and predictors of early training response

**DOI:** 10.1186/s12984-018-0433-4

**Published:** 2018-11-08

**Authors:** Christian Werner, Rebekka Rosner, Stefanie Wiloth, Nele Christin Lemke, Jürgen M. Bauer, Klaus Hauer

**Affiliations:** 1 Department of Geriatric Research, Agaplesion Bethanien Hospital Heidelberg, Geriatric Center at the Heidelberg University, Heidelberg, Germany; 2 Center for Geriatric Medicine, Heidelberg University, Heidelberg, Germany; 3 Department of Radiological Diagnostics, Theresien Hospital Mannheim, Mannheim, Germany; 4 Institute of Gerontology, Heidelberg University, Heidelberg, Germany; 5 Network of Aging Research (NAR), Heidelberg University, Heidelberg, Germany

**Keywords:** Dementia, Exergaming, Interactive, Dual-task, Postural control, Balance, Response, Cognition

## Abstract

**Background:**

Some studies have already suggested that exergame interventions can be effective to improve physical, cognitive, motor-cognitive, and psychological outcomes in patients with dementia (PwD). However, little is known about the training volume required to induce such positive effects and the inter-individual differences in training response among PwD. The aim of the study was to analyze the time course of changes in motor-cognitive exergame performances during a task-specific training program and to identify predictors of early training response in PwD.

**Methods:**

Secondary analyses of data from the intervention group (IG) of a randomized, placebo-controlled trial to improve motor-cognitive performances in PwD. Fifty-six geriatric patients with mild-to-moderate dementia randomized to the IG underwent a 10-week, task-specific training program (2×/week) on an exergame-based balance training system (Physiomat®), combining postural control tasks with cognitive tasks of an established neuropsychological test (Trail Making Test). Main outcome was the time required to complete different Physiomat®-Tasks (PTs) assessed at baseline (T1), training session 7 (TS7) and 14 (TS14), and post-intervention after 20 training sessions (T2). Reliable change indices were used to identify early responders from T1 to TS7. A multivariate logistic regression analysis was performed to determine independent predictors of early training response.

**Results:**

Completion time significantly improved already from T1 to TS7 in all PTs (*p* ≤ .001–.006), with moderate to very large effect sizes (*r* = .38–.52; Cohen’s d = .85–1.45). For most PTs, significant progressive improvements from TS7 to TS14 and TS14 to T2 were not observed. Thirty-one (59.6%) participants were classified as early responders and 21 (40.4%) as non-early responders. Lower baseline exergame performance and lower visuospatial and divided attention abilities were independently associated with early training response.

**Conclusions:**

Substantial task-specific improvements in complex motor-cognitive exergame performances can be obtained within a surprisingly short intervention period in PwD. Our results confirm that not only an excellent training response can be achieved in this patient population, but also that more vulnerable patients with greater deficits in domain-specific cognitive functions associated with fall risk may even reap the most and fastest benefit from motor-cognitive exergame interventions.

**Trial registration:**

ISRCTN registry, ISRCTN37232817 (retrospectively registered on 04/02/2012).

## Background

Attention is the first non-memory cognitive domain to be affected in dementia [1]. Divided attention, which is part of attentional control and the ability that allows individuals to perform two tasks simultaneously (i.e. dual tasks), represents the most affected aspect of attention [2, 3]. Under dual-task conditions, patients with dementia (PwD) or older people with cognitive impairment showed significantly reduced physical functions such as muscle strength [4], gait performance [5], and postural control [6, 7] compared to cognitively healthy older adults. Because everyday life involves many dual-task situations (e.g. walking while talking to someone) and deficits in dual-tasking have been associated with functional decline [8, 9] and falls [10, 11]**,** it has been suggested to incorporate dual-task exercises into preventive or rehabilitative training programs [12–14].

### Exergaming

Exergaming represents an emerging and unique form of dual-task training [15, 16], combining physical exercise with cognitively-challenging tasks in an interactive game-based way. In contrast to more traditional motor-cognitive dual-task exercises that combine distinct training tasks (e.g. walking while counting backwards), exergaming typically involves cognitive challenges directly embedded within the physical body movements that need to be performed to complete the game tasks projected onto a display screen [16]. The use of exergames in physical exercise and rehabilitation programs is progressively expanding as their playful character might help to encourage older people to participate in physical activity and to enhance their motivation toward exercise adherence [17, 18].

### Potential benefits of exergaming

Recent systematic reviews have shown positive effects of exergaming on physical, cognitive, dual-task and psychosocial outcomes in cognitively healthy older adults [19–26]. Given the growing evidence of its beneficial effects, increasing attention has recently been paid to exergaming also in older people with cognitive impairment and PwD. Some studies have already suggested that exergame interventions can be effective to improve balance and/or gait [27–29], motor-functional status and exercise capacity [30], global cognitive functioning and/or domain-specific cognitive functions (e.g. memory, attention, visuospatial and constructional abilities) [31–33], fear of falling [28, 29], depressive symptoms [31], and exergame performances in these populations [34–36] (for review, see also [37]).

### Time course of exergame-induced benefits

General dose-response relationships for traditional physical exercise suggest that untrained individuals may experience significant training benefits from low intensities, frequencies, and/or durations with small increases already during the early training period; however, with increasing performance of individuals, the magnitude of benefit may become less for a similar increase in intensity or amount of activity in the following training period [38, 39]**.** Although such knowledge is highly relevant for clinicians and practitioners to design time-efficient training protocols, little is yet known about dose-response relationships between exergaming (e.g. total duration of training period, number of training sessions) and its beneficial effects in older people with and without cognitive impairment or PwD [23, 40, 41]. Most frequently, previous studies did not include multiple assessment tests during the intervention period to address this research gap, but tested their participants only before and after the exergame-based training program, with intervention periods ranging widely from 1 to 24 weeks [28–35] (for review, see also [19–26]). Few studies conducted in older patients with Parkinson’s disease and healthy elderly [42, 43], older patients with chronic obstructive pulmonary disease [44], older adults with depression [45], or older community-dwelling fallers [46] used mid-term tests (at week 3–6) to assess physical and psychological outcomes after 30% or 50% of the total exergame intervention period (6–12 weeks, 2–5×/week, 30–40 min) [42, 45, 46] or investigated the intersession progression in exergame performances during a 7-week intervention period (2×/week) [43]. Most frequently, these studies observed significant improvements in study outcomes (e.g. balance, depressive symptoms, exergame performance) already after 2 to 4 weeks [42, 43, 46]. To our knowledge, in PwD, a similar study design including not only pre- and post-intervention assessments has been used only in two studies [27, 36]. Padala et al. [27], demonstrated significant balance improvements at post-intervention after 8 weeks but not at the mid-term of their exergame intervention after 4 weeks (5×/week, 30 min); and Fenney and Lee [36], who addressed the intersession changes of exergame performance during a 9-week intervention period (1×/week, 60 min), only described case reports, with heterogeneous results among cases (i.e. both improvements and deteriorations during the early training period), making it difficult to interpret their findings. In all these studies, training-induced changes in outcomes were always evaluated only relative to the pre-intervention assessment. None of them addressed comparisons between mid-term and post-intervention assessments [27, 42, 44–46], nor did they compare other assessment sessions during the intervention period among each other [43], which might have provided an even more detailed insight into the time course of changes in outcomes.

### Predictors of exergame training response

In people with cognitive impairment and PwD, effective exergame-based intervention studies have most frequently reported only main effects or mean group differences in changes without addressing inter-individual variability for their outcomes. However, individual differences and the identification of factors associated with training response has high clinical relevance [47]. For example, some persons may respond more favorably to an exergame intervention or the duration of the intervention period necessary to produce significant benefits may differ between persons with specific characteristics. To our knowledge, only Schwenk et al. [28] analyzed predictors of training response to a 4-week exergame-based balance training program in people with cognitive impairment (2×/week, 45 min), suggesting that low baseline performance and a history of falls were associated with greater improvements in balance.

### Previous work

We recently evaluated a 10-week, task-specific, motor-cognitive training program with an interactive, exergame-based balance training system (Physiomat®) in PwD. Results of our randomized controlled trial (RCT) demonstrated that, compared to a non-specific, motor placebo activity (unspecific, low-intensity strength and flexibility exercises for the upper body while seated), an exergame intervention significantly improved motor-cognitive performances of PwD in trained and untrained exergame tasks, with partly sustainable effects up to 3 months after training cessation [35]. Training-induced changes during the intervention period and predictors of early training response have not been addressed in this study.

### Study aims and hypotheses

The primary aim of these secondary analyses was to provide a more detailed insight into the time course of improvements in motor-cognitive exergame performances during a task-specific training program in patients with mild-to-moderate dementia. A secondary aim was to identify predictive factors associated with early training response. Based on general dose-response relationships for physical exercise [38, 39] and previous findings on predictors of exergame training response in people with cognitive impairment [28], we hypothesized that (1) time course of improvements in exergame performances would also be asymptotic, with the greatest training gains occurring during the early training period and (2) patients with the lowest initial performance would benefit most and the fastest from the exergame intervention.

## Methods

### Study design

This study presents secondary analyses of a double-blinded, randomized, placebo-controlled intervention trial on the effects of a dementia-specific motor-cognitive training program in patients with mild-to-moderate dementia. Details about the design, intervention, and main analyses of the RCT have been described previously [35, 48, 49]. The secondary analyses involved the motor-cognitive exergame performance of the intervention group (IG) on the Physiomat®, which was assessed at baseline before training (T1), at training session 7 (TS7) and 14 (TS14), and at post-intervention after 20 training sessions (T2) to analyze changes in the Physiomat® performance over the time course of the study period. Baseline patient characteristics were analyzed as potential predictors of early training response from T1 to TS7 for the Physiomat® performance.

### Study population

The process of screening, recruitment, enrollment and randomization of participants has been previously described in detail [35]. Inclusion criteria were as follows: ≥ 65 years; Mini-Mental Status Examination (MMSE, [50]) score 17–26; diagnosis of probable dementia based on comprehensive neuropsychological testing (Consortium to Establish a Registry for Alzheimer’s Disease [CERAD] test battery, [51]); ability to walk at least 10 m without a walking aid; no severe neurologic, cardiovascular, metabolic, or psychiatric disorders; residence within 15 km of the study center, and written informed consent.

### Intervention

**Fig. 1:**
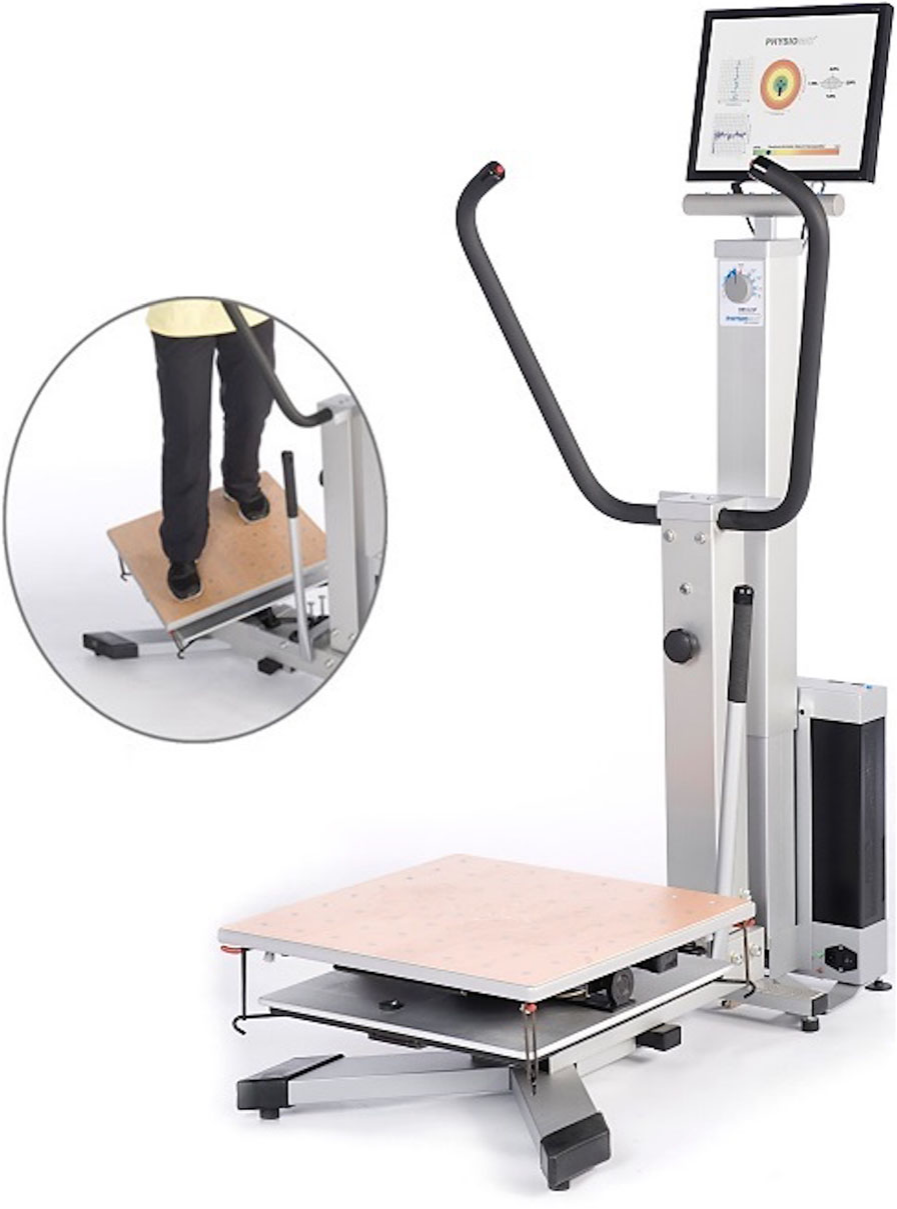
Exergame-based balance training system (Physiomat®). To solve a Physiomat® game task shown on a computer screen, the player must control and move the cursor by bending, tilting, and rotation movements while standing on the balance plat-form movable in the sagittal, frontal, and transversal plane (©EPL MEDIZINTECHNIK 2018 October 5, with kind permission from EPL MEDIZINTECHNIK)

Participants took part in an interactive, exergame-based training on the Physiomat® for 10 weeks (2×/week à 10 min; 20 sessions in total) supervised by a qualified trainer experienced in training PwD. The Physiomat® includes a balance platform moveable in the sagittal, frontal, and transversal plane. By platform-integrated displacement sensors, the three-dimensional movements of the platform are recorded and translated into linear movement of a cursor displayed on a 17-in. computer screen (Fig. 1). To solve a Physiomat® game task shown on the screen, the player must control and move the cursor by bending, tilting, and rotation movements while standing on the platform. A dementia-specific, patient-centered training approach (e.g. verbal instructions and cueing, haptic assistance, verbal praise) [52, 53] was used to train participants in performing a Physiomat®-Follow The Ball Task (FTBT) and more cognitively challenging Physiomat®-Trail Making Tasks (PTMTs) on five different complexity levels as defined by the number of digits to be connected (i.e. 4, 7, 9, 14, or 20 digits). The PTMTs were based on an established neuropsychological test (‘Zahlen-Verbindungs-Test’ = ‘Number-Connection-Test’ of the Nuremberg Age Inventory, TMT-NAI, [54], which is a modified version of the Trail Making Test validated for use in PwD.

Participants were instructed to move the cursor on the screen by weight shifting on the platform while holding onto the Physiomat® handles to follow a moving ball on the screen (FTBT) or to connect the digits in ascending order (PTMTs) as fast as possible (for further details on the intervention, see [35]). According to the participants’ individual performance level, the complexity of the Physiomat® tasks was successively increased as training progressed. The decision point for increasing the complexity was the ability to complete a Physiomat® task safely without assistance.

### Measurements

#### Outcome variable

The main outcome variable of this study was the Physiomat® performance assessed as the time required to complete the Physiomat® tasks (duration), which was directly derived from the data stream of the Physiomat®-integrated sensors during the gameplay [55]. The tasks included the same FTBT and PTMTs trained during the intervention period, and the same instructions were given as used for the Physiomat® training. No cueing or haptic assistance was provided by test administrator during the assessment. Each test session was started with the FTBT. After successfully completing the FTBT, the PTMT level was successively increased during the assessment until the participant was no longer able to complete a level. Participants performed two trials for each Physiomat® task, where the trial with the shortest duration was used for statistical analysis.

#### Descriptive and predictor variables

Demographic and clinical characteristics including age, gender, education, comorbidity (number of diagnoses, medications), recent history of falls (previous year), social status (community-dwelling vs. institutionalized), and physiological status for depression (Geriatric Depression Scale, 15-item version [56]) and fear of falling (Falls Efficacy Scale-International, 7-item version [57]) were documented from patient charts or by standardized patient interview.

Motor-functional status was measured by the Performance-Oriented Mobility Assessment (POMA, [58]), the Timed Up and Go [59], and the 5-chair stand test [60].

Cognitive status was screened using the MMSE [50]. Domain-specific cognitive functions were assessed by the CERAD test battery [51], including subtests of verbal fluency, visual naming (Boston Naming Test), verbal episodic memory encoding (word list memory), recall (word list recall) and recognition (word list recognition), visuospatial ability (constructional praxis), non-verbal episodic memory (constructional recall), and phonemic fluency; the modified Trail Making Test (TMT-NAI, [54]) for speed of information processing; and a Digit-Span Test (DST-NAI, [54]) for working memory. To assess global cognitive functioning, the demographically corrected CERAD total score (CERAD-TS) was calculated [61].

Motor-cognitive dual-task performance (divided attention) was assessed using a simultaneous walking and working memory task. Participants walked along a 5.79 m long GAITRite® instrumented walkway (CIR Systems Inc., Havertown, PA, USA: 4.88 m active area; 120 Hz sampling rate) at a maximum pace while counting backwards as fast as possible in steps of three. Each walk was initiated and terminated 1 m before and after the walkway to account for acceleration and deceleration. To quantify the overall motor-cognitive dual-task performance, the relative dual-task costs (DTC = ([dual task – single task]/single task × 100, [62]) of gait speed (DTC_gait speed_) and calculation steps (DTC_counting_) were combined (DTC_combined_ = (DTC_gait speed_ + DTC_counting_)/2). The dual-task test procedure and data processing as well as the biometrical quality of this dual-task assessment have been previously described in detail [63].

Initial training adherence from T1 to TS7 was documented as the percentage of the first seven training sessions attended relative to the number of the maximum possible training sessions offered in this period in which the participant completed these seven training sessions.

### Statistical analysis

Descriptive data were presented as frequencies and percentages for categorical variables, and means, standard deviations (SD) and ranges or medians and ranges for continuous variables as appropriate. To identify significant differences in the Physiomat® performance between the individual test sessions (T1, TS7, TS14, T2), we used one-way repeated-measures analyses of variance (ANOVAs) or Friedman ANOVAs on ranks (for non-normally distributed data) with Bonferroni-adjusted post-hoc paired-samples t-tests and Wilcoxon signed-rank tests, respectively. Effect sizes for post-hoc comparisons were calculated as Cohen’s d for paired-samples t-tests (0.2 ≤ d < 0.5 = small, 0.5 ≤ d < 0.8 = moderate, 0.8 ≤ d < 1.3 = large, d ≥ 1.3 = very large effect) and as effect size r for the Wilcoxon signed-rank tests (0.1 ≤ *r* < 0.3 = small, 0.3 ≤ *r* < 0.5 = moderate, 0.5 ≤ *r* < 0.7 = large, *r* ≥ 0.7 = very large effect) [64, 65]. Bonferroni-adjusted *p*-values were reported for the post-hoc multiple comparisons.

Reliable change indices (RCIs [66, 67]) were computed for the duration of each Physiomat® task to identify early responders from T1 to TS7. The RCI can be used to evaluate whether a participant’s change in pre- and post-intervention scores is beyond that which might be due to random measurement error, considering the instrument reliability and the sample variability specific for the population of interest. Based on data (i.e. test-retest reliabilities [r_tt_]; standard deviations of test [SD_1_] and retest [SD_2_]) of our previously published study on the biometrical quality of the Physiomat® assessment in PwD [55], the RCI for each Physiomat® task was calculated as follows: (1) standard error of measurement (SEM_1/2_ = SD_1/2_ × √[1 – r_tt_]); (2) standard error of the difference (S_diff_ = √[SEM_1_^2^ + SEM_2_^2^]), and (3) reliable change index (RCI = 1.96 × S_diff_). The calculated RCIs (FTBT: ± 9.5 s; PTMT level 1: ± 5.0 s, level 2: ± 7.5 s, level 3: ± 9.1, level 4: ± 11.0 s, level 5: ± 17.7 s) were used to determine whether a participant’s improvement in a specific Physiomat® task was sufficiently large to yield confidence that it was not due to measurement error. Taking into account the participant’s individual Physiomat® performance at baseline and the different complex Physiomat® tasks, an early responder (ER) was defined as a participant with an individual decrease in the duration after TS7 that exceeded the RCI either (1) for the most complex Physiomat® task completed at T1 or (2) for at least 50% of the Physiomat® tasks completed at T1. All other participants were defined as “non-early responders” (NER). To identify potential predictive factors associated with early training response, univariate analyses examined differences in participant baseline characteristics between ER and NER using unpaired t-tests, Mann-Whitney U-tests, and χ^2^ tests as appropriate. Independent baseline variables included demographic characteristics, comorbidity, psychological and motor-functional status, history of falls, global and domain-specific cognitive functioning, dual-task performance, baseline Physiomat® performances, and initial training adherence (Table 2). For the subsequent analysis, global cognitive functioning was defined by the CERAD-TS, which is regarded as being superior to scores of simplified screening measures such as the MMSE [68], and the baseline Physiomat® performance was defined by the FTBT duration, as the FTBT was the only Physiomat® task with baseline data available for all participants. Variables that showed significant differences in the univariate analyses were entered in a multivariate logistic regression analysis (likelihood ratio-based forward stepwise method) to determine independent predictors of early training response. Results of the regression model were reported as odds ratios (ORs) with 95% confidence intervals (CIs). The goodness-of-fit of the regression model was assessed by the Hosmer-Lemeshow test, and the amount of variance explained by model was expressed as Nagelkerke R^2^. Prediction accuracy of the model was defined as the percentage of correctly classified ERs and NERs. A two-sided *p*-value of ≤ .05 indicated statistical significance. Statistical analyses were performed using IBM SPSS Statistics for Windows, Version 23.0 (IBM Corp., Armonk, NY, USA).

## Results

### Sample characteristics

The sample for the secondary analyses included the 56 RCT participants allocated to the IG. Participants were multimorbid older patients with mild-to-moderate dementia and impaired motor-functional status (Table 1). Forty-five participants completed all four Physiomat® test sessions (T1, TS7, TS14, T2) over the study period. Four participants dropped out before TS7 due to serious medical events (*n* = 2, 3.6%) or lack of motivation (*n* = 2, 3.6%), and another seven dropped out during the later course of the intervention due to lack of motivation (*n* = 4, 7.1%), injurious falls (*n* = 2, 3.6%), and death (*n* = 1, 1.8%). Participants who dropped out did not differ significantly from those who stayed in the study for any descriptive variable at baseline (*p* = .327–.909) or any parameter for effects of intervention (*p* = .235–.933).

**Table 1:** Sample characteristics

Variable	Total sample (*n* = 56)
Age, years	82.7 ± 6.2 [65–94]
Females	39 (69.6)
Mini-Mental State Examination, score	22.2 ± 2.8 [17–26]
Education, years	11 [7–20]
Diagnoses	7.7 ± 3.8 [1–18]
Medications	7.6 ± 3.4 [0–14]
Taking cholinesterase inhibitors or memantine	13 (23.2)
Timed Up and Go, s	14.6 [6.5–52.7]
Performance Oriented Mobility Assessment, score	22.4 ± 4.3 [9–28]
5-chair stand test, s	14.8 ± 7.6 [6.8–39.1]
Geriatric Depression Scale, score	2 [0–9]
Falls Efficacy Scale-International, score	8.5 [7–19]
Fall in the previous year	23 (41.1)
Living situation	
Community-dwelling	39 (69.6)
Institutionalized	17 (30.4)

Data are presented as mean ± SD [range], n (%), or median [range]

### Time course of Physiomat® performance

**Fig. 2:**
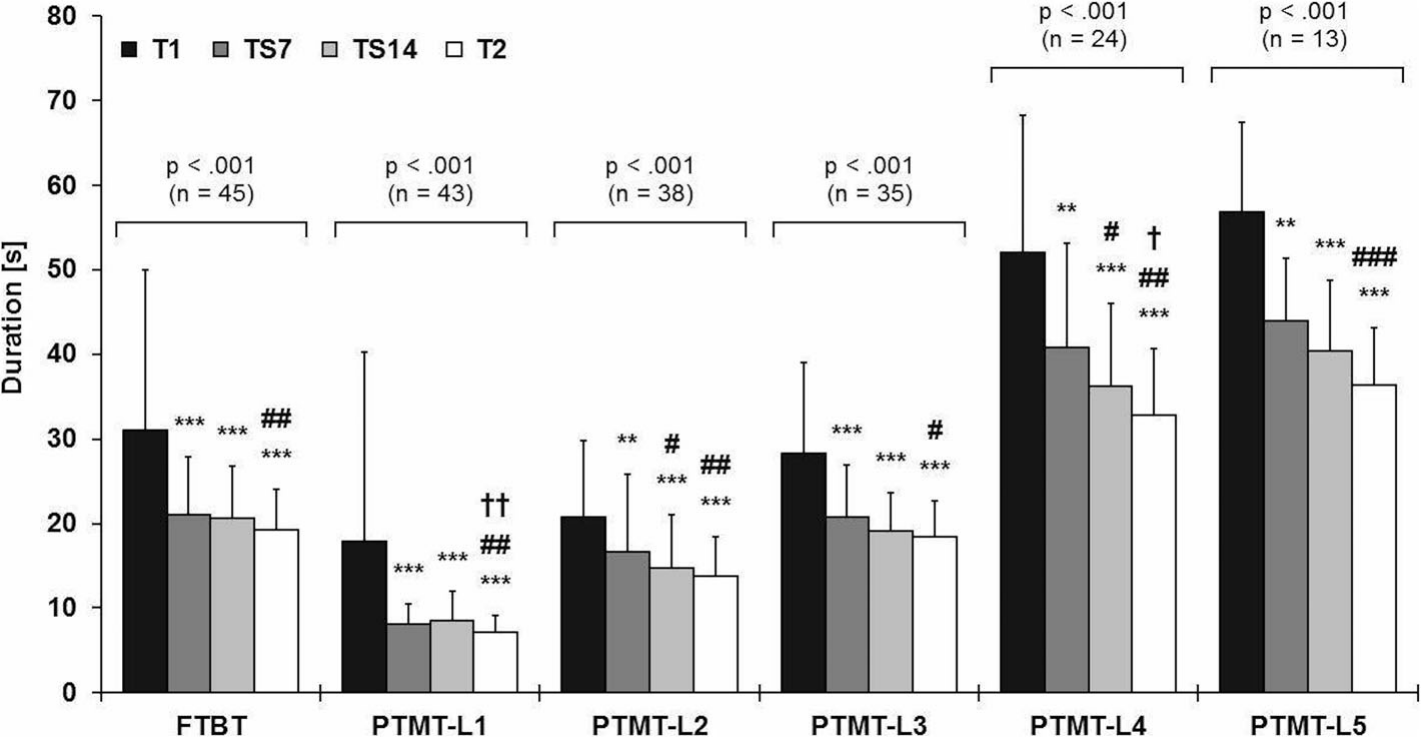
Performance in the different Physiomat® tasks at baseline (T1, black bars), training session 7 (TS7, dark gray bars) and 14 (TS14, light gray bars), and post- intervention (T2, white bars). Data are given as mean ± SD. FTBT, Physiomat®-Follow The Ball Task; PTMT, Physiomat®-Trail Making Task; L1–5, level 1–5. *P*-values are given for one-way repeated-measures ANOVAs (PTMT level 3–5) or Friedman ANOVAs on ranks (FTBT, PTMT level 1 & 2). Post-hoc multiple comparisons between the individual test sessions were performed with Bonferroni-adjusted paired t-tests (PTMT level 3–5) or Wilcoxon signed-rank tests (FTBT, PTMT level 1 & 2). Key to statistics: * *p* < .05, ** *p* < .01, *** *p* < .001, in comparison to T1; # *p* < .05, ## *p* < .01, ### *p* < .001, in comparison to TS7; † *p* < .05, †† *p* < .01, in comparison to TS14. Decrease in the duration (in seconds) indicates improvement in the Physiomat® performance

All 45 participants who stayed in the study successfully completed the rather low cognitively challenging FTBT at all four test sessions; however, the increasing difficulty of the Physiomat® tasks led to gradually decreasing samples sizes for the other, more complex PTMTs (level 1: *n* = 43 to level 5: *n* = 13, Fig. 2) as the participants reached their performance limit at individually different Physiomat® tasks.

Significant differences in the Physiomat® performance between the individual test sessions were found for all Physiomat® tasks (*p* < .001). Post-hoc analyses revealed significant improvements from T1 to TS7 in all Physiomat® tasks (*p* ≤ .001–.006), with moderate to very large effect sizes (FTBT, PTMT level 1 & 2: *r* = .38–.52; PTMT level 3–5: d = .85–1.45). For 4 out of 6 Physiomat® tasks, the Physiomat® performance did not significantly change between TS7 and TS14 (*p* = .359–.999). During this intermediate phase of the intervention period, significant positive intervention effects were observed only in the PTMT level 2 (*p* = .035) and level 4 (*p* = .026), with moderate effect sizes (level 2: *r* = .32; level 4: d = .68). From TS14 to T2, the Physiomat® performance significantly improved only in the PTMT level 1 (*p* = .009) and level 4 (*p* = .016). Effect sizes for these significant improvements were moderate (level 1: *r* = .34; level 4: d = .74). For all four other Physiomat® tasks, no significant improvements (*p* = .153–.999) were found during this late phase of the intervention period (TS14 to T2). For each Physiomat® task, the largest effect size between two consecutive test sessions was observed from T1 to TS7 (e.g. PTMT level 3: T1 to TS7: d = .85; TS7 to TS14: d = .34; TS14 to T2: d = .22). Over the last two thirds of the intervention period (TS7 to T2), the Physiomat® performance significantly improved across all Physiomat® tasks (*p* ≤ .001–.036), with moderate to very large effect sizes (FTBT, PTMT level 1 & 2: *r* = .34–.39; PTMT level 3–5: d = .53–1.34) and the largest effect sizes for the most complex Physiomat® tasks (PTMT level 4 & 5: d = 1.28–1.34).

### Predictors of early training response

**Fig. 3:**
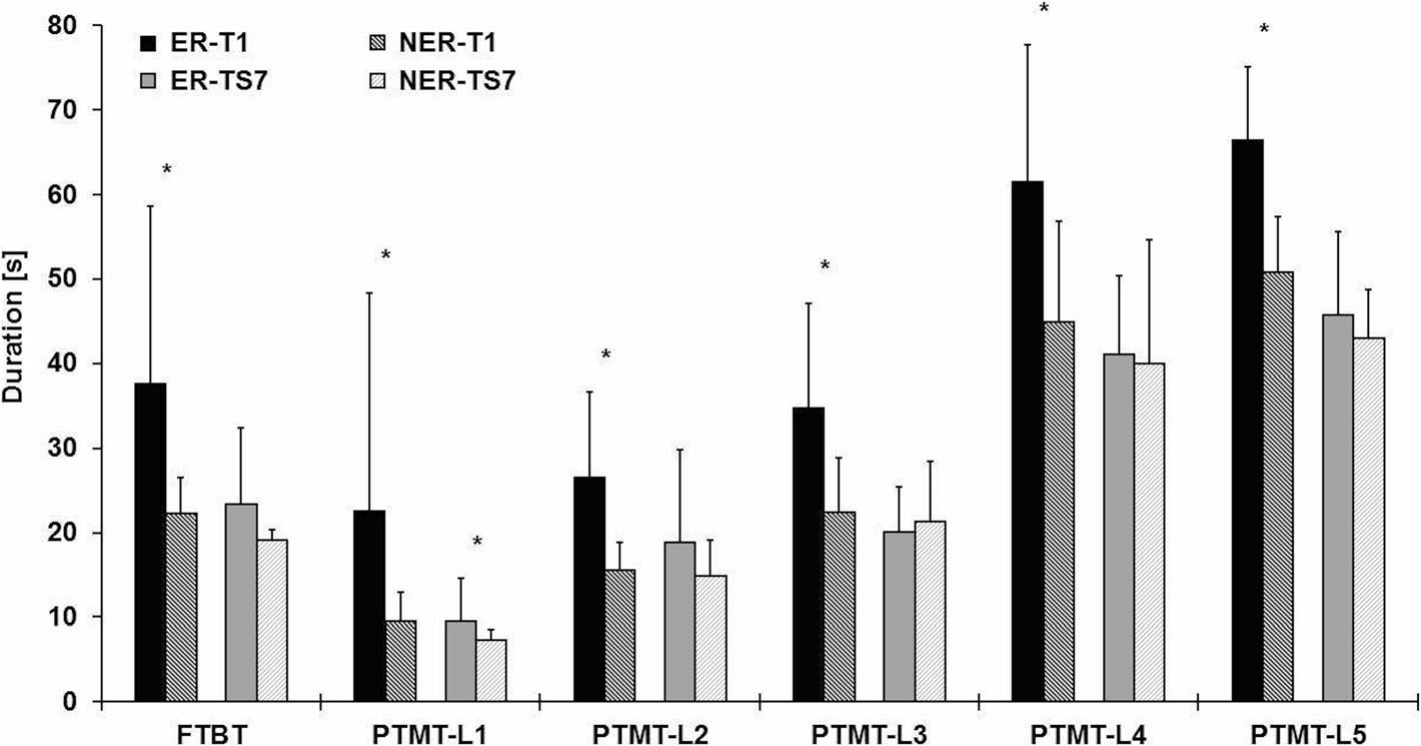
Performance in the different Physiomat tasks at baseline (T1) and training session 7 (TS7) for early responders (ER) and non-early responders (NER). Data are given as mean ± SD. FTBT, Physiomat®-Follow The Ball Task; PTMT, Physiomat®-Trail Making Task; L1–5, level 1–5. Paired-samples t-tests (PTMT-L3-L5) or Wilcoxon signed-rank tests (FTBT, PTMT-L1/L2) were performed to test differences between ERs and NERs at T1 and TS7, respectively. Key to statistics: * *p* < .05. Decrease in the duration (in seconds) indicates improvement in the Physiomat® performance

Potential predictive factors associated with early training response were analyzed in all the participants who completed the Physiomat® test sessions at T1 and TS7 (*n* = 52). Based on the definition of early training response by the RCIs across the different Physiomat® tasks, 31 (59.6%) participants were classified as ERs and 21 (40.4%) as NERs. The changes of ERs and NERs from T1 to TS7 within the individual Physiomat® tasks were presented in Fig. 3, suggesting that ERs showed lower baseline performance with higher improvements during this early training period up to a performance level at TS7 similar to those of the NERs. At post-intervention (T2), no significant differences in the Physiomat® tasks were found between ERs and NERs (*p* = .101–.911).

Comparisons between the baseline characteristics of two subgroups revealed that ERs initially showed significantly lower Physiomat® performances (FTBT: *p* < .001; PTMT: *p* ≤ .001–.036) as already indicated in Fig. 3, lower global cognitive functioning (MMSE: *p* = .039; CERAD-TS: *p* = .026), lower visuospatial ability (constructional praxis: *p* = .006), lower speed of information processing (TMT-NAI: *p* = .001), and lower dual-task performance (DTC_combined_: *p* = .040) than NERs (Table 2).

**Table 2:** Baseline comparisons between participant characteristics for early responders and non-early responders

Variable	ERs (*n* = 31)	NERs (*n* = 21)	*p*-value
Age, years^a^	83.5 ± 6.5 [65–93]	82.3 ± 6.0 [70–94]	.514
Females^b^	23 (74.2)	14 (66.7)	.557
Mini-Mental State Examination, score^a^	21.4 ± 2.8 [17–26]	23.0 ± 2.4 [17–26]	.039
Diagnoses^a^	7.5 ± 3.4 [1–17]	7.5 ± 4.1 [1–17]	.994
Medications^a^	7.3 ± 3.3 [0–13]	8.1 ± 3.6 [0–14]	.419
Timed Up and Go, s^c^	14.8 [9.8–52.7]	13.9 [6.5–51.2]	.714
Performance Oriented Mobility Assessment, score^a^	22.2 ± 4.0 [12–28]	23.1 ± 3.9 [15–28]	.405
5-chair stand test, s	14.8 ± 7.6 [6.8–39.1]	13.5 ± 5.1 [7.2–29.4]	.522
Geriatric Depression Scale, score^c^	2 [0–9]	2 [0–8]	.799
Falls Efficacy Scale-International, score^c^	8 [7–13]	8 [7–19]	.826
Fall in the previous year	13 (41.9)	8 (38.1)	.782
CERAD scores^a^			
Total score	70.7 ± 8.7 [54–87]	77.2 ± 12.0 [54–87]	.026
Verbal fluency	9.7 ± 3.7 [3–17]	11.3 ± 3.5 [4–18]	.124
Boston Naming Test	9.9 ± 2.7 [5–15]	11.0 ± 2.4 [5–14]	.150
Word list memory	10.5 ± 3.1 [4–16]	11.2 ± 3.8 [5–16]	.484
Word list recall	2.1 ± 1.8 [0–6]	2.0 ± 1.8 [0–5]	.900
Word list recognition	6.1 ± 2.7 [1–10]	7.1 ± 2.4 [2–10]	.172
Constructional praxis	7.0 ± 2.3 [2–11]	8.7 ± 1.8 [6–11]	.006
Constructional recall	1.7 ± 2.1 [0–7]	2.3 ± 2.2 [0–6]	.337
Phonemic fluency	6.8 ± 3.1 [0–15]	6.7 ± 4.0 [0–16]	.900
TMT-NAI, s^c^	114 [35–300]	57 [32–300]	.001
DST-NAI score^a^	8.6 ± 1.3 [6–11]	9.2 ± 1.0 [7–11]	.098
Dual-task performance (DTC_combined_)_,_ %^a^	−36.1 ± 22.3 [− 82.5- -0.4]	−24.0 ± 16.8 [− 54.0- -0.2]	.040
Baseline Physiomat® performance, s			
FTBT^c^	37.6 [18.5–121.1]	21.2 [17.1–31.5]	< .001
PTMT-L1^c,d^	15.6 [5.8–136.1]	8.2 [5.3–19.8]	< .001
PTMT-L2^c,e^	24.7 [10.3–57.3]	15.8 [10.5–21.5]	< .001
PTMT-L3^a,f^	34.7 ± 12.4 [21.6–64.5]	22.4 ± 6.4 [13.2–37.6]	< .001
PTMT-L4^a,g^	61.5 ± 16.2 [37.8–91.4]	44.9 ± 12.0 [31.6–67.5]	.007
PTMT-L5^a,h^	66.4 ± 8.7 [52.6–75.1]	53.7 ± 10.7 [39.1–76.9]	.036
Training adherence at TS7^a^	81.1 ± 20.3 [42.9–100]	76.7 ± 20.9 [33.3–100]	.451

Data are presented as mean ± SD [range], n (%), or median [range]. ERs, early responders; NERs, non-early responders; CERAD, Consortium to Establish a Registry for Alzheimer’s Disease, TMT-NAI, Trail Making Test from the Nuremberg Age Inventory; DST-NAI, Digit-Span Test from the Nuremberg Age Inventory; DTC_combined_, combined dual-task costs (i.e. [motor + cognitive dual-tasks costs]/2); FTBT, Physiomat®-Follow The Ball Task; PTMT, Physiomat®-Trail Making Task; L1–5, level 1–5. *P*-values for ^a^t-tests, ^b^χ^2^ test, and ^c^Mann-Whitney U-tests. *P*-values in bold indicate statistical significance (*p* ≤ .05). Comparison between ^d^*n* = 30 ERs vs. *n* = 20 NERs, ^e^*n* = 27 ERs vs. *n* = 18 NERs, ^f^*n* = 21 ERs vs. *n* = 18 NERs; ^g^*n* = 12 ERs vs. *n* = 13 NERs, ^h^*n* = 5 ERs vs. *n* = 9 NERs

When the variables significantly associated with early training response were entered into the multivariate logistic regression model, baseline Physiomat® performance (OR = 1.261, *p* = .003), constructional praxis (OR = 0.558, *p* = .019), and dual-task performance (OR = 0.943, *p* = .031), were identified as independent predictors of early training response (Table 3). The regression model was significant (χ^2^ = 33.96, df = 3, *p* < .001) and demonstrated goodness-of-fit (Hosmer-Lemeshow-Test: χ^2^ = 5.45, df = 8, *p* = .709). It accounted for 64.8% of variance in training response (Nagelkerke R^2^ = .648) and correctly classified 84.6% participants as ERs or NERs.

**Table 3:** Multivariate logistic regression model for predictors of early training response

Variable	β	SEM	OR (95% CI)	*P-*value
Constructional praxis^a^	−.583	.248	.558 (.344–.907)	.019
Baseline Physiomat® performance^b^	−.232	.079	1.261 (1.081–1.471)	.003
Dual-task performance^c^	−.058	.027	.943 (.895–.995)	.031

Removed from model: TMT-NAI (*p* = .745), CERAD total score (*p* = .565). ^a^Lower scores indicate lower constructional praxis ability; ^b^higher scores indicate lower baseline Physiomat® performance; ^c^lower scores indicate lower dual-task performance

## Discussion

To the best of our knowledge, the presented study is the first (1) to provide a detailed insight into the time course of changes in motor-cognitive exergame performances during a task-specific training program and (2) to examine potential predictive factors that are associated with early training response in the vulnerable population of multimorbid older patients with mild-to-moderate dementia and impaired motor-functional status.

### Time course of Physiomat® performance

Deficits in motor-cognitive dual-tasking has been repeatedly identified as predictor for functional decline [8, 9] and falls [10, 11], and dual-task abilities have been reported to be reduced in PwD [5–7]. One of the major findings of this study was that in PwD exergame-based dual-task performances can be substantially improved after a surprisingly short period of task-specific interactive motor-cognitive training. We observed significant improvements in all Physiomat® tasks already after 3 weeks.

In older patients with Parkinson’s disease and healthy elderly, significant improvements in some but not all Nintendo Wii Fit™ exergames have also been reported after similar durations of task-specific training (2–3 weeks) [43]. In PwD, only a usability study was found, showing that exergame performances can be improved after 4 weeks of task-specific training [34]. However, the frequency and total amount of exercise during the intervention period in this study were substantially higher (4 × 60 min/week = 240 min), and the exergame intervention covered interactive video-sports games with considerably less complex concurrent motor-cognitive tasks (Nintendo Wii Sports™ Bowling and Tennis), compared to our study with a training protocol of two 10 min-exergame sessions per week (3 × 20 min/week = 60 min) on an exergame-based balance platform combining whole-body postural control tasks with complex cognitive tasks of an established neuropsychological test (TMT-NAI, [54]). The prompt training-induced effects on such interactive motor-cognitive dual-task performances have not been previously reported in PwD. These findings suggest that an exergame-based motor-cognitive training program has the potential to represent a time-efficient intervention for improving movement control under cognitive load in PwD, which may contribute to reduced fall risk in this highly vulnerable patient population [14]. Such interactive motor-cognitive training programs might also be associated with more general effects on global cognitive functioning, as previously reported for exergaming [32] or interactive visuomotor game training [69] in cognitively impaired older adults after 10- to 14-week training periods. Based on our findings that improvements in task-specific exergame performances can be made after a much shorter training period, future studies should assess whether potential transfer effects related to exergame interventions can be achieved also after short intervention periods, or even assess the time course of such potential transfer effects.

The time course of improvements in exergame performances seemed to be asymptotic rather than linear, with the greatest part of the total training effects already apparent after the early, 3-week intervention phase (T1 to TS7) across all Physiomat® tasks. After these prompt initial improvements at TS7, the positive effect of the intervention decreased and participants seemed to reach a training plateau during the intermediate intervention phase given the chosen training frequency and intensity. For most of the Physiomat® tasks, the extension of the intervention period by another two training intervals of 3 weeks did not result in significant progressive improvements compared to the corresponding previous test session (TS7 vs. TS14, TS14 vs. T2). However, taking this two training intervals together (TS7 to T2 = 6 weeks), the performance in all Physiomat® tasks significantly improved over the last two thirds of the intervention period. This time course of exergame performances confirmed our hypothesis and is in line with established exercise training principles and general dose-response relationships reported for exercise training [38, 39], suggesting that in training novices and untrained individuals with high potential for improvements, a low training dosage (intensity, frequency, duration) with small increases may already be sufficient to induce significant improvements after a short training period; however, as training duration continues and the performance of individuals increases, the rate of improvements begins to slow down for a similar increase in intensity or amount of activity in the following training period. Thus, a higher rate in the increase of the training parameters (intensity, frequency, and/or duration) is required to provide further effective training stimuli. For each Physiomat® task, we observed significant improvements after the initial intervention phase (T1 to TS7), which in turn may have decreased the participant’s potential for improvements within the tasks during the subsequent training period. To achieve further training gains from the increased performance level and this test session on, a training intensification in the following training period seems to have been necessary. The frequency, i.e. the number of training sessions per week, could not be increased in our study due to its design, but we were able to successively further increase the intensity, i.e. the complexity level of the Physiomat® tasks, as participants improved and training progressed. However, for some participants, a higher rate in increase of the intensity was not possible, as the number of available complexity levels of the Physiomat® tasks was limited. For example, the intensity for high-preforming participants that reached the most complex Physiomat® tasks during the early training period could only be further increased by instructions to complete the most complex Physiomat® tasks even more quickly. This rather low rate of further increase in intensity for these participants may have also affected the impact of the intervention during the subsequent training period. Overall, our approach for increasing the intensity might not have been sufficient to induce the same significant improvements observed for the initial 3-week training interval (T1 to TS7) also for the consecutive training intervals of equal length (TS7 to TS14, T14 to T2). Rather, it seemed that the extension of the training duration (TS7 to T2 = 6 weeks) might have played a more important role to further improve participants’ performance beyond that reached after the early intervention phase. This finding is also consistent with the idea of the general dose-response principle for exercise training [38], indicating that improvements in some health-related variables may be more related to the volume and amount of exercise than to its intensity.

Over the last two thirds of the intervention period (TS7 to T2) the largest effects were observed for the highest PTMT levels. This might be related to their higher complexity and to the greater sensitivity to detect changes that has been previously reported for these Physiomat® tasks [35]. For the less complex Physiomat® tasks, potential ceiling effects for those participants who completed the higher PTMT levels already at baseline and possibly reached their maximum performance level on the less complex Physiomat® tasks already after the initial training period (T1 to TS7) may have affected the impact of the extended training duration. In the highest PTMT levels, however, there may still have been a higher potential for these high-performing participants to further improve during the subsequent training period, even after initial significant training response, due to the higher complexity of these tasks.

### Predictors of early training response

Previous studies in older people with cognitive impairment or PwD identified low baseline performance in primary outcomes (i.e., balance, maximal strength, and/or motor-functional performance) to be independently predictive for positive training response to physical exercise or exergame interventions [28, 52, 70]. Supporting our hypothesis, based on these previous findings, the regression analysis of our study revealed similar results for exergame-based motor-cognitive performances, such that participants with lower initial performance in these outcomes were those who experienced the greatest training gains over the first 3 weeks of the intervention period. The inverse relationship between the magnitude of benefits and the baseline status for an initial training phase was also described in the general dose-response principles for exercise training [38, 39], indicating that those individuals with the lowest performance benefit the most and the fastest from an exercise intervention.

Some studies have found that lower global cognitive functioning negatively influence training response to physical exercise interventions [71–75], whereas other studies have not [28, 52, 70, 76, 77]. Interestingly, and in contrast to all these studies, more severe global cognitive impairment was initially associated with early training response in the univariate analysis of our study. However, the multivariate analysis suggested that this association might actually be explained by the lower performance in domain-specific cognitive functions, whose effects on trainability have rarely been examined in previous studies [52, 76], rather than by the level of global cognitive functioning, which was not independently associated with early training response in our regression model. In particular, the multivariate analysis revealed that lower visuospatial ability and divided attention were independent predictors of early training response. It is conspicuous that lower performance in these cognitive subdomains was identified to be predictive for early training response as spatial orientation and divided attention were stated to be required and trained on the Physiomat® [35]. Because of their greater deficits in these task-related cognitive functions, ERs may have had more room to improve during the early training period than NERs.

In the univariate analysis, lower performance in speed of information processing as assessed by the TMT-NAI was initially associated with early training response; however, this association was lost in the multivariate analysis, maybe because it was captured by other model covariates, especially by the baseline performance on the Physiomat®, whose game tasks were based also on the TMT-NAI.

Results confirm that not only an excellent training response can be achieved despite cognitive impairment, but also that those participants with greater deficits in training-related, domain-specific cognitive functions of visuospatial ability and divided attention, which both have been associated with fall risk [10, 78], benefit the most and the fastest from the exergame intervention.

### Limitations and future research

The present study has some limitations. First, the exergame performance of the control group was only assessed before and at the end of the intervention period. Therefore, the time course of the effects of the exergame intervention in the IG could not be compared to those of the non-specific, motor placebo activity. However, in our previous RCT, we have already demonstrated that improvements in the IG over the entire study period are specifically related to the exergame intervention, with no significant improvements in the control group for exergame-related performances [35]. Second, the increasing difficulty of the Physiomat® tasks during the test sessions led to gradually decreasing sample sizes across the tasks. The statistical analyses of the more complex tasks may therefore be limited by a small sample size. By using increasing difficulty levels for assessment, however, it was ensured that each participant could have been adequately challenged to assess his/her individual maximum performance level ('testing the limits') and to prevent ceiling and floor effects at all test sessions. Third, our results are restricted to patients with mild-to-moderate dementia and so cannot be generalized to those with more severe dementia. Fourth, the small sample size may have affected the results of the regression analysis and limited the precious of our conclusions. Fifth, according to the preplanned study design, potential transfer effects of the exergame intervention (e.g. on balance, cognitive functioning) were not analyzed in this study, representing potential targets for future research.

## Conclusions

The present study reveals for the first time that substantial task-specific improvements in complex motor-cognitive performances can be achieved after a surprisingly short exergame-based training program in patients with mild-to-moderate dementia. According to general dose-response relationships for physical exercise, our findings demonstrate that the rate of improvements induced by the exergame intervention decreases as training progresses and that with increasing performance level of individuals a longer training period is required to achieve further improvements. Current findings also highlight the trainability and rehabilitation potential of PwD, especially for more vulnerable patients with low initial performance and more severe impairments in training-related cognitive functions who may even reap the most and fastest benefit from motor-cognitive exergame interventions.

## Abbreviations

ANOVA: Analysis of variance
CERAD: Consortium to Establish a Registry for Alzheimer’s Disease
CERAD-TS: Total score of the CERAD test battery
CI: Confidence interval
DST-NAI: Digit-Span Test of the Nuremberg Age Inventory
DTC: Dual-task cost
ER: Early responder
FTBT: Physiomat®-Follow The Ball Task
IG: Intervention group
MMSE: Mini-Mental State Examination
NER: Non-early responder
OR: Odds ratio
POMA: Performance Oriented Mobility Assessment
PTMT: Physiomat®-Trail Making Task
PwD: Patients with dementia
RCI: Reliable change index
RCT: Randomized controlled trial
r_tt_: Test-retest reliability
SD: Standard deviation
SEM: Standard error of measurement
T2: Post-intervention
TMT-NAI: ‘Number-Connection-Test’ (Trail Making Test) of the Nuremberg Age Inventory
TS14: Training session 14
TS7: Training session 7

## References

[1] Perry RJ, Hodges JR (1999). Attention and executive deficits in Alzheimer’s disease. A critical review Brain.

[2] Baddeley AD, Baddeley HA, Bucks RS, Wilcock GK (2001). Attentional control in Alzheimer’s disease. Brain.

[3] Perry RJ, Watson P, Hodges JR (2000). The nature and staging of attention dysfunction in early (minimal and mild) Alzheimer’s disease: relationship to episodic and semantic memory impairment. Neuropsychologia.

[4] Hauer K, Marburger C, Oster P (2002). Motor performance deteriorates with simultaneously performed cognitive tasks in geriatric patients. Arch Phys Med Rehabil.

[5] Muir SW, Speechley M, Wells J, Borrie M, Gopaul K, Montero-Odasso M (2012). Gait assessment in mild cognitive impairment and Alzheimer’s disease: the effect of dual-task challenges across the cognitive spectrum. Gait Posture.

[6] Hauer K, Pfisterer M, Weber C, Wezler N, Kliegel M, Oster P (2003). Cognitive impairment decreases postural control during dual tasks in geriatric patients with a history of severe falls. J Am Geriatr Soc.

[7] Manckoundia P, Pfitzenmeyer P, d’Athis P, Dubost V, Mourey F (2006). Impact of cognitive task on the posture of elderly subjects with Alzheimer’s disease compared to healthy elderly subjects. Mov Disord.

[8] Lundin-Olsson L, Nyberg L, Gustafson Y (1998). Attention, frailty, and falls: the effect of a manual task on basic mobility. J Am Geriatr Soc.

[9] Faulkner KA, Redfern MS, Rosano C, Landsittel DP, Studenski SA, Cauley JA (2006). Reciprocal influence of concurrent walking and cognitive testing on performance in older adults. Gait Posture..

[10] Muir-Hunter SW, Wittwer JE (2016). Dual-task testing to predict falls in community-dwelling older adults: a systematic review. Physiotherapy.

[11] Beauchet O, Annweiler C, Dubost V, Allali G, Kressig RW, Bridenbaugh S (2009). Stops walking when talking: a predictor of falls in older adults?. Eur J Neurol.

[12] Schwenk M, Lauenroth A, Oster P, Hauer K, Braumann K-M, Stiller N (2010). Effektivität von körperlichem Training zur Verbesserung motorischer Leistungen bei Patienten mit demenzieller Erkrankung. Bewegungstherapie bei internistischen Erkrankungen.

[13] Ghai S, Ghai I, Effenberg AO (2017). Effects of dual tasks and dual-task training on postural stability: a systematic review and meta-analysis. Clin Interv Aging.

[14] Montero-Odasso M, Speechley M (2018). Falls in cognitively impaired older adults: implications for risk assessment and prevention. J Am Geriatr Soc.

[15] Monteiro-Junior RS, Vaghetti CA, Nascimento OJ, Laks J, Deslandes AC (2016). Exergames: Neuroplastic hypothesis about cognitive improvement and biological effects on physical function of institutionalized older persons. Neural Regen Res.

[16] Tait JL, Duckham RL, Milte CM, Main LC, Daly RM (2017). Influence of sequential vs. simultaneous dual-task exercise training on cognitive function in older adults. Front Aging Neurosci.

[17] van Diest M, Lamoth CJ, Stegenga J, Verkerke GJ, Postema K (2013). Exergaming for balance training of elderly: state of the art and future developments. J Neuroeng Rehabil.

[18] Molina KI, Ricci NA, de Moraes SA, Perracini MR (2014). Virtual reality using games for improving physical functioning in older adults: a systematic review. J Neuroeng Rehabil..

[19] Stanmore E, Stubbs B, Vancampfort D, de Bruin ED, Firth J (2017). The effect of active video games on cognitive functioning in clinical and non-clinical populations: a meta-analysis of randomized controlled trials. Neurosci Biobehav Rev.

[20] Schoene D, Valenzuela T, Lord SR, de Bruin ED (2014). The effect of interactive cognitive-motor training in reducing fall risk in older people: a systematic review. BMC Geriatr.

[21] Skjaeret N, Nawaz A, Morat T, Schoene D, Helbostad JL, Vereijken B (2016). Exercise and rehabilitation delivered through exergames in older adults: an integrative review of technologies, safety and efficacy. Int J Med Inform.

[22] Laufer Y, Dar G, Kodesh E (2014). Does a Wii-based exercise program enhance balance control of independently functioning older adults? A systematic review. Clin Interv Aging.

[23] Chao YY, Scherer YK, Montgomery CA (2015). Effects of using Nintendo Wii exergames in older adults: a review of the literature. J Aging Health.

[24] Ogawa EF, You T, Leveille SG (2016). Potential benefits of exergaming for cognition and dual-task function in older adults: a systematic review. J Aging Phys Act.

[25] Taylor LM, Kerse N, Frakking T, Maddison R (2018). Active video games for improving physical performance measures in older people: a meta-analysis. J Geriatr Phys Ther.

[26] Li J, Theng YL, Foo S (2016). Effect of exergames on depression: a systematic review and meta-analysis. Cyberpsychol Behav Soc Netw.

[27] Padala KP, Padala PR, Malloy TR, Geske JA, Dubbert PM, Dennis RA (2012). Wii-fit for improving gait and balance in an assisted living facility: a pilot study. J Aging Res.

[28] Schwenk M, Sabbagh M, Lin I, Morgan P, Grewal GS, Mohler J (2016). Sensor-based balance training with motion feedback in people with mild cognitive impairment. J Rehabil Res Dev.

[29] Padala KP, Padala PR, Lensing SY, Dennis RA, Bopp MM, Roberson PK (2017). Home-based exercise program improves balance and fear of falling in community-dwelling older adults with mild Alzheimer’s disease: a pilot study. J Alzheimers Dis.

[30] Ben-Sadoun G, Sacco G, Manera V, Bourgeois J, Konig A, Foulon P (2016). Physical and cognitive stimulation using an exergame in subjects with normal aging, mild and moderate cognitive impairment. J Alzheimers Dis.

[31] Gonzalez-Palau F, Franco M, Bamidis P, Losada R, Parra E, Papageorgiou SG (2014). The effects of a computer-based cognitive and physical training program in a healthy and mildly cognitive impaired aging sample. Aging Ment Health.

[32] Yamaguchi H, Maki Y, Takahashi K (2011). Rehabilitation for dementia using enjoyable video-sports games. Int Psychogeriatr.

[33] Weybright E, Dattilo J, Rusch F (2010). Effects of an interactive video game (Nintendo Wii) on older women with mild cognitive impairment. Ther Recreat J.

[34] Legouverneur G, Pino M, Boulay M, Rigaud A-S (2011). Wii sports, a usability study with MCI and Alzheimer’s patients. Alzheimers Dement.

[35] Wiloth Stefanie, Werner Christian, Lemke Nele Christin, Bauer Jürgen, Hauer Klaus (2017). Motor-cognitive effects of a computerized game-based training method in people with dementia: a randomized controlled trial. Aging & Mental Health.

[36] Fenney A, Lee TD (2010). Exploring spared capacity in persons with dementia: what WiiTM can learn. Act Adapt Aging.

[37] van Santen J, Droes RM, Holstege M, Henkemans OB, van Rijn A, de Vries R (2018). Effects of exergaming in people with dementia: results of a systematic literature review. J Alzheimers Dis.

[38] Haskell WL (1994). J.B. Wolffe Memorial Lecture. Health consequences of physical activity: understanding and challenges regarding dose-response. Med Sci Sports Exerc.

[39] Hoffmann J (2002). Physiological aspects of sport training and performance.

[40] Manera V, Ben-Sadoun G, Aalbers T, Agopyan H, Askenazy F, Benoit M, et al. Recommendations for the use of serious games in neurodegenerative disorders: 2016 Delphi panel. Front Psychol. 2017;8(1243). 10.3389/fpsyg.2017.01243 PMC5524915 28790945

[41] Manlapaz DG, Sole G, Jayakaran P, Chapple CM (2017). A narrative synthesis of Nintendo Wii fit gaming protocol in addressing balance among healthy older adults: what system works?. Games Health J.

[42] Esculier JF, Vaudrin J, Beriault P, Gagnon K, Tremblay LE (2012). Home-based balance training programme using Wii fit with balance board for Parkinsons’s disease: a pilot study. J Rehabil Med.

[43] dos Santos Mendes FA, Pompeu JE, Modenesi Lobo A, Guedes da Silva K, Oliveira Tde P, Peterson Zomignani A (2012). Motor learning, retention and transfer after virtual-reality-based training in Parkinson’s disease-effect of motor and cognitive demands of games: a longitudinal, controlled clinical study. Physiotherapy.

[44] Albores J, Marolda C, Haggerty M, Gerstenhaber B, Zuwallack R (2013). The use of a home exercise program based on a computer system in patients with chronic obstructive pulmonary disease. J Cardiopulm Rehabil Prev.

[45] Rosenberg D, Depp CA, Vahia IV, Reichstadt J, Palmer BW, Kerr J (2010). Exergames for subsyndromal depression in older adults: a pilot study of a novel intervention. Am J Geriatr Psychiatry.

[46] Williams MA, Soiza RL, Jenkinson AM, Stewart A (2010). EXercising with computers in later life (EXCELL) - pilot and feasibility study of the acceptability of the Nintendo® WiiFit in community-dwelling fallers. BMC Res Notes.

[47] Chmelo EA, Crotts CI, Newman JC, Brinkley TE, Lyles MF, Leng X (2015). Heterogeneity of physical function responses to exercise training in older adults. J Am Geriatr Soc.

[48] Werner C, Wiloth S, Lemke NC, Kronbach F, Jansen CP, Oster P (2017). People with dementia can learn compensatory movement maneuvers for the sit-to-stand task: a randomized controlled trial. J Alzheimers Dis.

[49] Lemke NC, Werner C, Wiloth S, Oster P, Bauer JM, Hauer K. Transferability and sustainability of motor-cognitive dual-task training in patients with dementia: a randomized controlled trial. Gerontology. 2018:1–16. 10.1159/000490852. 10.1159/000490852 30041173

[50] Folstein MF, Folstein SE, McHugh PR (1975). “Mini-mental state”. A practical method for grading the cognitive state of patients for the clinician. J Psychiatr Res.

[51] Morris JC, Mohs RC, Rogers H, Fillenbaum G, Heyman A (1988). Consortium to establish a registry for Alzheimer’s disease (CERAD) clinical and neuropsychological assessment of Alzheimer’s disease. Psychopharmacol Bull.

[52] Hauer K, Schwenk M, Zieschang T, Essig M, Becker C, Oster P (2012). Physical training improves motor performance in people with dementia: a randomized controlled trial. J Am Geriatr Soc.

[53] Schwenk M, Oster P, Hauer K (2008). Kraft- und Funktionstraining bei älteren Menschen mit demetieller Erkrankung. Praxis Physiotherapie.

[54] Oswald WD, Fleischmann UM (1999). Das Nürnberger-alters-Inventar (NAI) - Testinventar & NAI-Testmanual und Textband.

[55] Wiloth S, Lemke N, Werner C, Hauer K (2016). Validation of a computerized, game-based assessment strategy to masure training effects on motor-cognitive functions in people with dementia. JMIR Serious Games.

[56] Sheikh JI, Yesavage JA, Brink TL (1986). Geriatric depression scale (GDS): recent evidence and development of a shorter version. Clinical gerontology: a guide to assessment and intervention.

[57] Hauer KA, Kempen GI, Schwenk M, Yardley L, Beyer N, Todd C (2011). Validity and sensitivity to change of the falls efficacy scales international to assess fear of falling in older adults with and without cognitive impairment. Gerontology.

[58] Tinetti ME (1986). Performance-oriented assessment of mobility problems in elderly patients. J Am Geriatr Soc.

[59] Podsiadlo D, Richardson S (1991). The timed “up & go”: a test of basic functional mobility for frail elderly persons. J Am Geriatr Soc.

[60] Guralnik JM, Simonsick EM, Ferrucci L, Glynn RJ, Berkman LF, Blazer DG (1994). A short physical performance battery assessing lower extremity function: association with self-reported disability and prediction of mortality and nursing home admission. J Gerontol.

[61] Chandler MJ, Lacritz LH, Hynan LS, Barnard HD, Allen G, Deschner M (2005). A total score for the CERAD neuropsychological battery. Neurology.

[62] Abernethy B (1988). Dual-task methodology and motor skills research: some applications and methodological constraints. J Hum Mov Stud.

[63] Lemke NC, Wiloth S, Werner C, Hauer K (2017). Validity, test-retest reliability, sensitivity to change and feasibility of motor-cognitive dual task assessments in patients with dementia. Arch Gerontol Geriatr.

[64] Cohen J (1988). Statistical power analysis for the behavioral sciences.

[65] Rosenthal JA (1996). Qualitative descriptors of strength of association and effect size. J Soc Serv Res.

[66] Jacobson NS, Truax P (1991). Clinical significance: a statistical approach to defining meaningful change in psychotherapy research. J Consult Clin Psychol.

[67] Iverson GL (2001). Interpreting change on the WAIS-III/WMS-III in clinical samples. Arch Clin Neuropsychol.

[68] Ehrensperger MM, Berres M, Taylor KI, Monsch AU (2010). Early detection of Alzheimer’s disease with a total score of the German CERAD. J Int Neuropsychol Soc.

[69] de Boer C, Echlin HV, Rogojin A, Baltaretu BR, Sergio LE. Thinking-while-moving exercises may improve cognition in elderly with mild cognitive deficits: a proof-of-principle study. Dement Geriatr Cogn Dis Extra. 2018:248–58. 10.1159/000490173 PMC6103359 30140274

[70] Schwenk M, Dutzi I, Englert S, Micol W, Najafi B, Mohler J (2014). An intensive exercise program improves motor performances in patients with dementia: translational model of geriatric rehabilitation. J Alzheimers Dis.

[71] Uemura K, Shimada H, Makizako H, Doi T, Yoshida D, Tsutsumimoto K (2013). Cognitive function affects trainability for physical performance in exercise intervention among older adults with mild cognitive impairment. Clin Interv Aging.

[72] Ghisla MK, Cossi S, Timpini A, Baroni F, Facchi E, Marengoni A (2007). Predictors of successful rehabilitation in geriatric patients: subgroup analysis of patients with cognitive impairment. Aging Clin Exp Res.

[73] Rösler A, Krause T, Niehuus C, von Renteln-Kruse W (2009). Dementia as a cofactor for geriatric rehabilitation-outcome in patients with osteosynthesis of the proximal femur: a retrospective, matched-pair analysis of 250 patients. Arch Gerontol Geriatr.

[74] Morghen S, Gentile S, Ricci E, Guerini F, Bellelli G, Trabucchi M (2011). Rehabilitation of older adults with hip fracture: cognitive function and walking abilities. J Am Geriatr Soc.

[75] Hershkovitz A, Kalandariov Z, Hermush V, Weiss R, Brill S (2007). Factors affecting short-term rehabilitation outcomes of disabled elderly patients with proximal hip fracture. Arch Phys Med Rehabil.

[76] Schwenk M, Zieschang T, Englert S, Grewal G, Najafi B, Hauer K (2014). Improvements in gait characteristics after intensive resistance and functional training in people with dementia: a randomised controlled trial. BMC Geriatr.

[77] Beloosesky Y, Grinblat J, Epelboym B, Weiss A, Grosman B, Hendel D (2002). Functional gain of hip fracture patients in different cognitive and functional groups. Clin Rehabil.

[78] Naslund John (2010). Visuospatial Ability in Relation to Fall Risk and Dementia. Archives of Neurology.

